# Transcriptome data sets of *Piscinibacter sakaiensis* grown in 702 complex medium, maltose, and PET plastic

**DOI:** 10.1128/mra.01466-25

**Published:** 2026-03-12

**Authors:** Jessica K. Novak, Jeffrey G. Gardner

**Affiliations:** 1 Department of Biological Sciences, University of Maryland Baltimore County 1068 , Baltimore, Maryland, USA; University of Southern California, Los Angeles, California, USA

**Keywords:** *Piscinibacter sakaiensis*, plastic degradation, transcriptomics

## Abstract

*Piscinibacter sakaiensis* 201-F6 is a gram-negative bacterium capable of metabolizing and fermenting poly(ethylene) terephthalate (PET) plastics and simple sugars. Here, we announce three transcriptome data sets of *Piscinibacter sakaiensis* grown in 702 complex medium, maltose, and PET plastic.

## ANNOUNCEMENT

Since the discovery and isolation of *Piscinibacter sakaiensis* 201-F6 in 2016 (formerly *Ideonella sakaiensis*), the poly(ethylene) terephthalate (PET)-degrading capabilities of the bacterium have been heavily studied (1–3). Specifically, the PETase and mono(2-hydroxyethyl) terephthalic acid-degrading (MHETase) enzymes have undergone extensive biochemical (4–7) and genetic characterization (8–10). However, fewer studies have characterized the physiological response of *P. sakaiensis* on PET and other fermentable carbon sources (9, 11). Therefore, we announce three transcriptomic data sets of *P. sakaiensis* grown in 702 complex medium, maltose, or PET plastic.

*P. sakaiensis* 201-F6 was purchased from the National Institute of Technology and Evaluation, Biological Resource Center (Tokyo, Japan), revived in 702 complex medium, and stored in 50% glycerol (wt/vol) at −80°C. Transcriptome sampling was conducted as previously described (12, 13). Briefly, the frozen cell stock was revived via colony isolation and propagation in 702 complex medium. Once *P. sakaiensis* cells grew to full density (optical density at 600 nm [OD_600_] ~1.5), a 1:100 dilution of cell culture was used to inoculate 300 mL flasks in biological triplicate with either 702 complex medium as the sole nutrient source, 702 medium with 0.5% (wt/vol) maltose, or 702 medium with 1% (wt/vol) PET plastic at 30°C with high aeration (200 RPM). The PET plastic was derived from a Deer Park water bottle and autoclaved in a 30-min dry cycle (121°C and 16 psi) prior to growth experiments to ensure sterility. Growth was tracked via optical density (OD_600_) measurements, and sampling occurred at ~7 h for cells grown in 702 medium, ~9 h in 702 medium with maltose, and ~27 h in 702 medium with PET plastic when the cells were most likely to begin utilizing the plastic (10). Once each desired OD_600_ was reached, 35 mL of culture was aseptically collected and added to 5 mL phenol:ethanol (5:95, vol/vol) to stop metabolism. The metabolically stopped cell suspensions were then immediately centrifuged at 8,000 × *g* for 5 min at 4°C. Supernatants were removed, and cell pellets were flash-frozen in a dry ice and ethanol bath for 5 min before storage in −80°C and shipment to GeneWiz (Plainfield, NJ) for RNA extraction, library preparation, and sequencing.

RNA was extracted from cell pellets using the Qiagen RNeasy Mini Kit (Hilden, Germany), and RNA sequencing library preparation was performed using the NEBNext Ultra II RNA Library Preparation Kit for Illumina (NEB, Ipswich, MA) according to the manufacturer’s instructions. The sequencing libraries were assessed for quality using the Agilent Tapestation 4200 (Agilent Technologies, Palo Alto, CA) and quantified using a Qubit 4.0 Fluorometer (Thermo Fisher Scientific, Waltham, MA), as well as by quantitative PCR (KAPA Biosystems, Wilmington, MA). The sequencing libraries were multiplexed and clustered onto a flow cell on the Illumina HiSeq 4000 according to the manufacturer’s instructions. The samples were sequenced using a 2 × 150 bp paired-end configuration that produced an average of 56,917,716 reads per sample (Table 1).

**TABLE 1:** Quality overview of sequenced transcriptomes

Sample identities	No. of reads^*a*^	Mean quality score	Bases (%) *Q* > 30^*b*^	SRA accession no.
702 Rep 1	53,838,115	35.34	90.6	SRX31399924
702 Rep 2	66,104,127	35.31	90.5	SRX31399925
702 Rep 3	52,944,756	35.30	90.5	SRX31399926
702 + Maltose Rep 1	70,199,158	35.40	90.9	SRX31399927
702 + Maltose Rep 2	52,034,782	35.38	90.8	SRX31399928
702 + Maltose Rep 3	60,242,398	35.40	90.9	SRX31399929
702 + PET Rep 1	54,468,132	35.18	90.0	SRX31399930
702 + PET Rep 2	54,066,645	35.33	90.6	SRX31399931
702 + PET Rep 3	48,361,328	35.25	90.3	SRX31399932

^*a*^ 150 bp read length.

^*b*^ Percent of base calls with a quality (*Q*) score of >30.

## Data Availability

The raw sequence data may be found under NCBI BioProject number PRJNA1377810 and NCBI SRA ID SRP653582.
